# State- or trait-like individual differences in dream recall: preliminary findings from a within-subjects study of multiple nap REM sleep awakenings

**DOI:** 10.3389/fpsyg.2015.00928

**Published:** 2015-07-06

**Authors:** Serena Scarpelli, Cristina Marzano, Aurora D’Atri, Maurizio Gorgoni, Michele Ferrara, Luigi De Gennaro

**Affiliations:** ^1^Department of Psychology, University of Rome “Sapienza”, RomeItaly; ^2^Department of Life, Health and Environmental Sciences, University of L’Aquila, L’AquilaItaly

**Keywords:** dreaming, theta oscillations, REM sleep, frontal cortex, episodic memory

## Abstract

We examined the question whether the role of EEG oscillations in predicting presence/absence of dream recall (DR) is explained by “state-” or “trait-like” factors. Six healthy subjects were awakened from REM sleep in a within-subjects design with multiple naps, until a recall and a non-recall condition were obtained. Naps were scheduled in the early afternoon and were separated by 1 week. Topographical EEG data of the 5-min of REM sleep preceding each awakening were analyzed by power spectral analysis [Fast Fourier Transform (FFT)] and by a method to detect oscillatory activity [Better OSCillations (BOSC)]. Both analyses show that REC is associated to higher frontal theta activity (5–7 Hz) and theta oscillations (6.06 Hz) compared to NREC condition, but only the second comparison reached significance. Our pilot study provides support to the notion that sleep and wakefulness share similar EEG correlates of encoding in episodic memories, and supports the “state-like hypothesis”: DR may depend on the physiological state related to the sleep stage from which the subject is awakened rather than on a stable individual EEG pattern.

## Introduction

Recent neuroimaging studies on dreaming underlined the continuity between mechanisms involved in mental activity across sleep and wakefulness (De Gennaro et al., 2011; Eichenlaub et al., 2014a,b). Some support to this general notion comes from a recent EEG study (Marzano et al., 2011). Although the results of EEG studies are heterogeneous (Nir and Tononi, 2010), they show a link between the alpha band (8–12 Hz) and the retrieval of sleep mentation from non-rapid eye movement (NREM) sleep (Takeuchi et al., 2003; Esposito et al., 2004; Chellappa et al., 2009, 2011; Marzano et al., 2011; Ruby et al., 2013; Eichenlaub et al., 2014a).

Moreover, Marzano et al. (2011) found an increase of frontal theta activity (5–7 Hz) prior to dream recall (DR) from REM sleep. The theta activity over the frontal areas seems particularly involved in the retrieval of episodic mnestic traces also in wakefulness (Hsieh and Ranganath, 2014; Scarpelli et al., 2015). For instance, Klimesch (1999) reported that the increase in theta power anticipates a subsequent successful performance in episodic memory encoding. Theta enhancement was also observed during retrieval of previously learnt information (Klimesch et al., 2000, 2006). Furthermore, some studies revealed that high pre-stimulus theta activity is associated to subsequent successful recall (REC; Addante et al., 2011; Gruber et al., 2013), supporting the view that memory performance depends on state-related physiological factors *before* the beginning of the task (Addante et al., 2011).

Hence, specific EEG topography and frequencies during sleep have been associated to presence/absence of DR, and they are also predictive of DR frequency. These EEG patterns are also related to episodic memory during wakefulness (Marzano et al., 2011). However, these findings did not respond to the question whether the EEG activity associated to DR was interpretable in terms of “state-like” or “trait-like” differences. In other terms, they fail to address the issue whether the EEG patterns predictive of DR depend on peculiar EEG oscillations of the specific physiological *scenario* from which subject is awakened (i.e., state-like) or whether these patterns represent a stable individual feature of the subjects (i.e., trait-like). Excluding a specific protocol (i.e., a 40-h multiple nap protocol), which introduces a chronobiological confound (Chellappa et al., 2009, 2011), *intraindividual* EEG differences in DR have been investigated only by *between-subjects* designs (Takeuchi et al., 2003; Esposito et al., 2004; Marzano et al., 2011; for a review see Scarpelli et al., 2015).

Another issue is that the standard method adopted to analyze EEG activity related to human cognition is the Fast Fourier Transform (FFT) analysis (van Vugt et al., 2007). This technique is mainly designed for stationary and regular signals (Mallat, 1998; Zhan et al., 2006; van Vugt et al., 2007). In other words, FFT analysis has a limited time-frequency resolution (Bruns, 2004). However, brain signals are seldom stationary (Whitten et al., 2011) and the EEG patterns correlated to dream contents retrieval are more likely characterized by oscillatory (non-stationary) activity. In this respect, Caplan et al. (2001) implemented an oscillatory detection method [Better OSCillation (BOSC)]. This well-established method, which takes into account the functional form of a “background” signal, is able to discriminate the segments of the recording that deviate significantly from the spectral characteristics of the background (Marzano et al., 2011; Whitten et al., 2011). The BOSC has been successfully applied to identify theta oscillations in the human neocortex during both sleep and wakefulness (Caplan et al., 2001, 2003; Caplan and Glaholt, 2007; Marzano et al., 2011, 2013), and delta oscillations in the hippocampal formation by stereo-EEG recordings (Moroni et al., 2012), associated with encoding and consolidation of declarative memory.

In this perspective article, we present a pilot study aimed to disentangle the “state-/trait-like” issue by investigating the role of EEG *oscillations* during REM sleep in DR by a *within-subjects* design.

## Methods

### Subjects

Six healthy right-handed subjects [5 females (F), 1 male (M); mean age = 21.25 ± 2.75] participated as paid volunteers. They were university students who were selected from the database of our laboratory. Participants were required to maintain regular sleep habits during the week preceding the experimental session: compliance was verified by sleep diaries, which each subjects had to fill out every morning within, 15-min after the awakening. The study was approved by the Institutional Ethics Committee of the Department of Psychology of University of Rome Sapienza and was conducted in accordance with the Declaration of Helsinki.

### Procedure

Each subject participated at least in two naps during the early afternoon, until we collected both a DR (REC) and a Non-Recall (NREC) condition. Actually, the mean number of naps was 3.5 (SD = 1.0; min = 2, max = 5). Each experimental session was separated by 1 week.

The polysomnographic (PSG) recordings (28 EEG channels, EMG, and EOG channels) were acquired in a sound-proof, temperature-controlled room. A Brain Amp system was used for PSG recordings. EEG signals were analogically high-pass filtered with a time constant of 0.3 s and low-pass filtered at 30 Hz. The 28 unipolar EEG derivations of the international 10–20 system (C3, C4, Cp1, Cp2, Cp5, Cp6, Cz, F3, F4, F7, F8, Fc1, Fc2, Fc5, Fc6, Fp1, Fp2, Fz, O1, O2, Oz, P3, P4, P7, P8, Pz, T7, T8) were recorded from scalp electrodes with averaged mastoid reference. The submental EMG was recorded with a time constant of 0.03 s. Bipolar horizontal eye movements were recorded with a time constant of 1 s. The bipolar horizontal electrooculogram (EOG) was recorded from electrodes placed about 1 cm from the medial and lateral canthi of the dominant eye. Impedance of these electrodes was kept below 5 k**Ω**.

Subjects were awakened from first REM sleep episode without stage shifts during the last 5-min of sleep. Hence, analyses have been carried out on 5-min intervals of uninterrupted and artifact-free REM sleep. Assignment to the condition and stage scoring was confirmed off-line.

After awakening, subjects filled out a sleep and dream diary. Preliminarly, each subject was instructed to consider any distinct mental activity occurring during sleep as a dream.

## Analyses

We have processed only the data obtained from subjects who have reported both REC and NREC conditions upon awakening from REM sleep.

### Data Processing and Statistics

The polygraphic signals of the 5-min of REM sleep preceding the awakening were analog-to-digital converted on-line with sampling rate of 250 Hz. Artifacts were rejected off-line on an 8 s basis by visual inspection. Only tonic REM sleep periods were included in the analysis, to avoid artifacts from rapid eye movements on EEG power.

Power EEG, based on FFT data, were divided in the canonical bands: delta (0.50–4.75 Hz), theta (5.00–7.75 Hz), alpha (8.00–11.75 Hz), sigma (12.00–15.75 Hz), and beta (16.00–24.75 Hz).

Statistical comparisons (paired *t*-tests) were computed on log-transformed values between REC and NREC conditions. The analyses were performed separately for each frequency band and each cortical electrode. EEG power maps were computed for statistical comparisons between REC and NREC conditions from REM sleep. To adjust the α-value for multiple comparisons, a Bonferroni correction was applied (Sankoh et al., 1997; Perneger, 1998). Considering the mean correlation between the dependent variables in REM sleep (*r* = 0.66) and the number of statistical comparisons (140 tests), α level was adjusted to 0.009 (*t* ≥ 4.12).

According to the specific aims of our study, we applied the BOSC analysis to the EEG signals in order to identify oscillatory activity (Caplan et al., 2001; Whitten et al., 2011). The analysis was performed separately for each frequency of interest (within the 0.50–24 Hz range) and each electrode. For a given episode, we defined an epoch longer than a duration threshold, DT (set to three cycles in our analysis) during which wavelet power at each frequency exceeded a power threshold (PT). This PT was chosen as follows in the 5 min segments: (1) The EEG was wavelet transformed (Morlet wavelet, window of six cycles) at 47 logarithmically spaced frequencies in the range 0.50–25 Hz. The average of the log-transform of these wavelet values yielded the wavelet power spectrum. (2) The background noise spectrum assumed the form Power(*f*) = Af–a. The estimate of this background has been obtained by fitting the observed spectrum (at each electrode) with a linear regression in log–log units. The background at f^∗^ has been estimated on the mean of its corresponding χ^2^(2) probability distribution function. The PT was set to the 95th percentile of the theoretical probability distribution. The proportion of time in which significant oscillations were detected within a 5 min sleep period was termed *P*_episode_ (Caplan et al., 2001, 2003; Caplan and Glaholt, 2007; van Vugt et al., 2007).

The BOSC analysis was performed on the EEG signals recorded from each scalp location during the last 5-min of REM sleep before awakening and was averaged across subjects. Then, *P*_episode_ values in correspondence of the theta peak frequency were compared between REC and NREC conditions by paired *t*-tests. Bonferroni correction (Sankoh et al., 1997; Perneger, 1998) was applied (*r* = 0.69; 28 tests) and α level was adjusted to 0.018 (*t* ≥3.46).

## Results

### EEG Power Spectra

**FIGURE 1:**
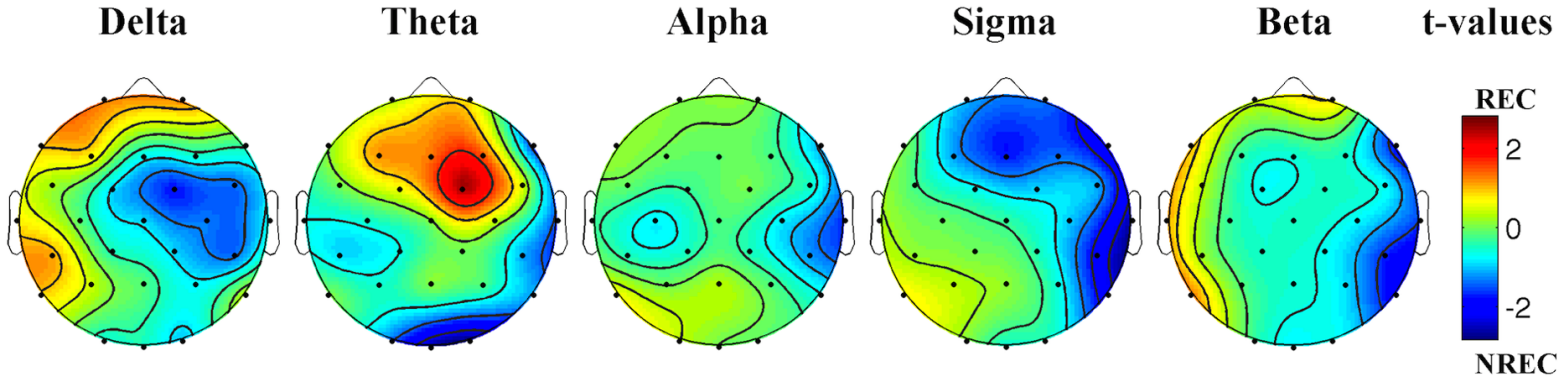
**Statistical maps reporting topographic EEG power differences, assessed by paired *t*-tests, between the REC and NREC conditions.** Values are expressed in terms of *t*-values: positive t values (warmer colors) indicate a prevalence of the REC over the NREC conditions and vice versa. The maps are based on 28 derivations (electrode positions indicated by black dots). Values are color coded and plotted at the corresponding position on the planar projection of the hemispheric scalp model. Values between electrodes were interpolated (biharmonic spline interpolation).

The statistical maps (**Figure 1**) show a predominance of theta activity over the right-frontal regions (Fc2) in the REC compared to NREC condition (*t* = 2.47, *p* = 0.065). Furthermore, maps show a lower theta power in centro-occipital regions (Oz, *t* = –2.69, *p* = 0.043; O2, *t* = –2.82, *p* = 0.037) and a lower temporal sigma activity in REC vs. NREC condition (T8, *t* = –2.64, *p* = 0.046). However, these comparisons are not statistically significant after the Bonferroni correction.

### Detection of Oscillatory Activity

**FIGURE 2:**
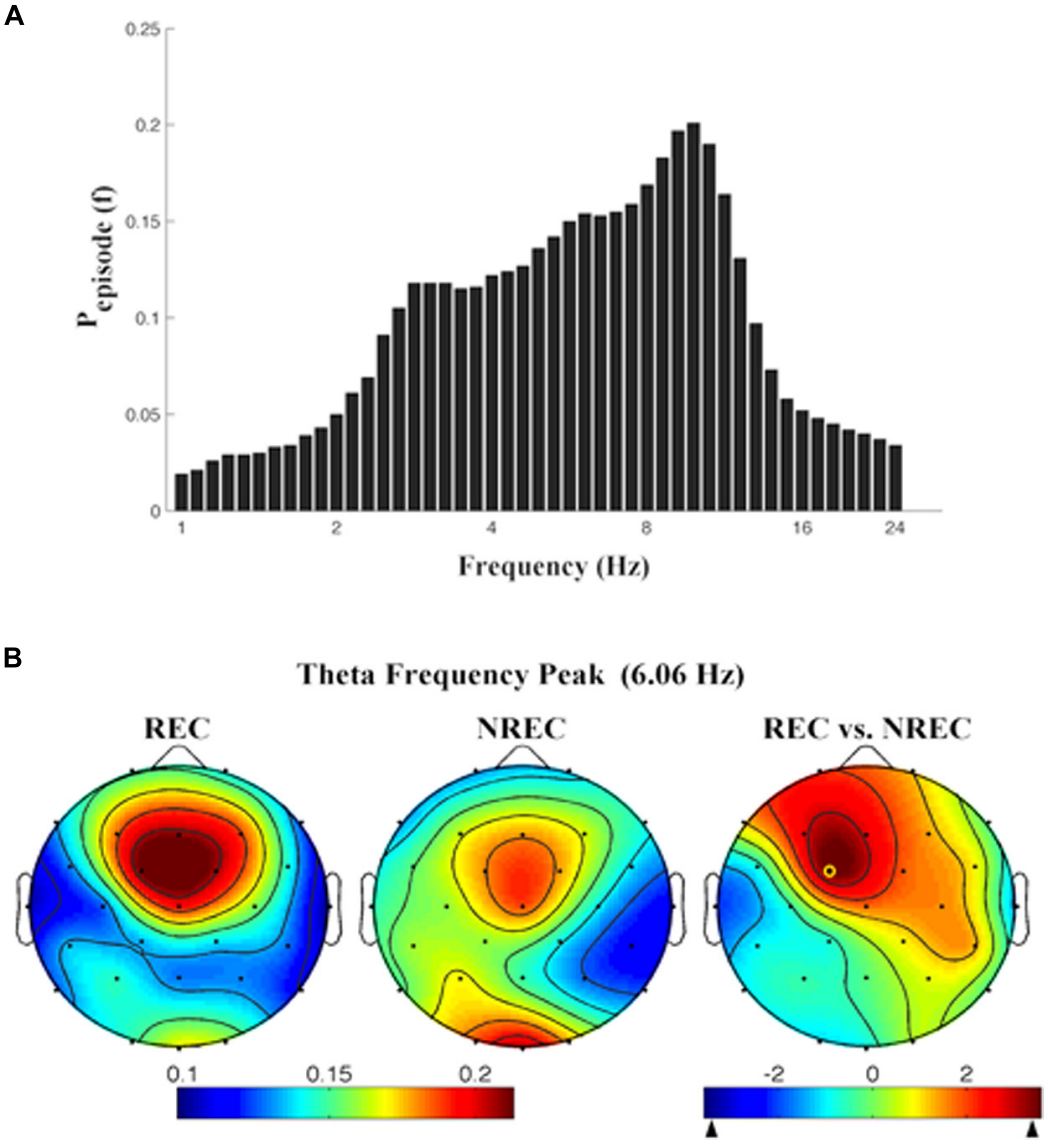
**(A)** Mean proportion of time [*P*_episode_ (*f*)] of EEG activity during last segment (5 min) of REM sleep during which oscillations were detected by the BOSC method at each frequency in the 0.25–25.00 Hz Individual oscillations detected across all frequencies by the BOSC method have been averaged across all subjects, and error bars denote SEM *P*_episodesEsp_ (*f*). **(B)** Topographic distribution of the frequency peak of theta oscillatory activity. From the left, the first two maps show the topographic distribution of the mean proportion of time in which oscillations were detected (*P*_episode_) in correspondence of the selected frequency of interest (6.06 Hz) in the 5-min intervals preceding awakening, in REC and NREC conditions respectively. The first map on the right side shows topographic statistical *P*_episode_ differences (assessed by paired *t*-tests) between REC and NREC conditions. The yellow circle indicates the electrode (Fc1, *t* = 3.60; *p* = 0.015), where the comparison between REC and NREC conditions is significant. The maps are based on 28 derivations (electrode positions indicated by black dots). Values are color-coded and plotted at the corresponding position on the planar projection of the hemispheric scalp model. Values between electrodes were interpolated (biharmonic spline interpolation).

As depicted by **Figure 2A**, which details EEG oscillations averaged across all the derivations, the EEG recordings of the pre-awakening segment shows three peaks: alpha band peaking at 9.85 Hz, theta band peaking at 6.06 Hz and delta band peaking at 2.83 Hz.

Given the association between frontal theta increase and DR obtained by FFT (Marzano et al., 2011), we calculated the topographic distribution of the peak frequency of the oscillatory theta activity (6.06 Hz; **Figure 2B**). In other words, we detail the topographic distribution of the mean proportion of time in which oscillations were detected (*P*_episode_) in correspondence of the peak frequency of interest (6.06 Hz) in the 5-min intervals preceding awakening, associated to REC and NREC conditions. The statistical map (**Figure 2B**) points to a robust difference between REC and NREC conditions, significant in correspondence of Fc1 (*t* = 3.60, *p* = 0.015). Although not significant after the correction, the differences in theta oscillations are also remarkable at Fz (*t* = 2.81, *p* = 0.037) and F3 (*t* = 2.78, *p* = 0.039), suggesting that the increase of theta oscillations in the REC condition spreads to most of the frontal area.

## Discussion

Our preliminary data are substantially coherent with previous findings, supporting the idea that higher frontal theta oscillations (and theta activity) are associated to a successful DR (Marzano et al., 2011).

Keeping in mind the relationship between frontal theta activity and memory processes (Hsieh and Ranganath, 2014; Scarpelli et al., 2015), if confirmed on a larger sample, these results may provide further support to the hypothesis that the mechanisms of encoding and retrieval of episodic memories remain the same across wakefulness and sleep (De Gennaro et al., 2011; Marzano et al., 2011). Moreover, our results provide arguments in favor of the “state-like hypothesis,” according to which the EEG correlates of DR depend on the physiological background of the sleep state in the segment closer to the time of dream report collection, rather than on stable interindividual EEG pattern characterizing each subject.

Notably, the difference between REC vs. NREC conditions is significant only for the BOSC analysis. In other words, although the topographical differences are substantially coherent across the FFT and BOSC analyses, nevertheless differences are larger when comparing theta oscillations. It is possible that this specific difference may be explained by the relatively small sample size. On the other hand, this finding may also suggest that the predictive relation between EEG and presence/absence of DR mostly depends on the oscillatory theta activity, more than on tonic (background) theta activity. This would strengthen the coherence of the present results with those obtained during wakefulness on the relation between theta oscillations and encoding/consolidation of episodic memory. It is worth noting that theta activity (as calculated by FFT routines) is also an expression of homeostatic processes during sleep (even during REM sleep: Marzano et al., 2010). Accordingly, changes in theta activity may index *both* homeostatic processes during sleep and functional relations with mechanisms of dreaming, and this could explain statistical differences between our different analyses. Clearly, at this stage of the study any other consideration would be (further) speculative. Only a larger sample size and the analysis of NREM awakenings (REC and NREC conditions for each subject) will allow drawing definitive conclusions.

## Conclusion and Future Directions

To the best of our knowledge, this is the first investigation carried out with the specific purpose to address the “state- or trait-like” issue in order to reveal the EEG correlates of DR. The nap protocol allowed us to collect several sleep recordings in the same time window aimed to obtain REC and NREC conditions in a within-subjects design, allowing a control for trait-like factors and circadian factors (Nielsen, 2004).

Hence, we have found an EEG pattern that appears different for the two conditions. Namely, our data provide information about the specific EEG activity which predicts *whether* subjects will REC a dream when they wake up from REM sleep. On the one hand, our preliminary results give some further support to the “continuity hypothesis” (Domhoff, 2003; Schredl, 2003, 2009) between neurophysiological mechanisms for the encoding and retrieval of episodic memory in sleep and wakefulness (Marzano et al., 2011). On the other hand, for the specific purpose of the study, our preliminary results suggest that DR is associated to a frontal theta oscillatory activity during the last segment of REM sleep before the awakening, supporting a “state-like hypothesis.”

As a further approach to this issue, future investigations should consider protocols with multiple awakenings during the night for each subject, and collect DR/NREC for each REM or NREM sleep cycle, also assessing possible homeostatic and circadian influences on DR.

Future studies should also extend the range of the considered EEG oscillations beyond the beta oscillations, since some studies found a functional coupling between fronto-temporal theta and gamma (25–40 Hz) activity in sleep and in wakefulness related to memory processes (Sederberg et al., 2003; Sauseng et al., 2009; Marshall et al., 2011). A significant increase of theta and gamma oscillations during wake encoding predicts the subsequent REC of episodic memory (Sederberg et al., 2003; Nyhus and Curran, 2010). Furthermore, it was reported that the transcranial direct current stimulation (tDCS) at 5 Hz increases the gamma oscillations during REM sleep (Marshall et al., 2011) and –more directly– that the stimulation on the fronto-temporal area in the gamma band during REM sleep elicits lucid dreams, defined as particular state of consciousness in which the sleeper is aware of his dreaming (Hobson et al., 2014; Voss et al., 2014).

## Author Contributions

Substantial contributions to the conception and design of the work: LDG, MF.

Acquisition, analysis of data: SS, ADA, MG, CM.

Interpretation of data: LDG, MF, SS, CM.

Drafting the work and revising it critically for important intellectual content: LDG, MF, SS, ADA, MG, CM.

Final approval of the version to be published: LDG, MF, SS, ADA, MG, CM.

Agreement to be accountable for all aspects of the work in ensuring that questions related to the accuracy or integrity of any part of the work are appropriately investigated and resolved: LDG, MF, SS, ADA, MG, CM.

## Conflict of Interest Statement

The authors declare that the research was conducted in the absence of any commercial or financial relationships that could be construed as a potential conflict of interest.
